# Some characters of bacterial cellulases in goats’ rumen elucidated by metagenomic DNA analysis and the role of fibronectin 3 module for endoglucanase function

**DOI:** 10.5713/ajas.20.0115

**Published:** 2020-08-21

**Authors:** Khanh Hoang Viet Nguyen, Trong Khoa Dao, Hong Duong Nguyen, Khanh Hai Nguyen, Thi Quy Nguyen, Thuy Tien Nguyen, Thi Mai Phuong Nguyen, Nam Hai Truong, Thi Huyen Do

**Affiliations:** 1 Institute of Biotechnology, Vietnam Academy of Science and Technology, Cau Giay, Ha Noi 100000, Vietnam; 2 Graduate University of Science and Technology, Vietnam Academy of Science and Technology, Cau Giay, Ha Noi 100000, Vietnam; 3 Biology Faculty, Hanoi University of Natural Science, Vietnam National University, Thanh Xuan, Ha Noi 100000, Vietnam; 4 School of Biotechnology and Food technology, Hanoi University of Science and Technology, Ha Noi 100000, Vietnam

**Keywords:** Cellulase Modularity, Goats’ Rumen, Metagenomic DNA Data, FN3 Module, Ig Module

## Abstract

**Objective:**

Fibronectin 3 (FN3) and immunoglobulin like modules (Ig) are usually collocated beside modular cellulase catalytic domains. However, very few researches have investigated the role of these modules. In a previous study, we have sequenced and analyzed bacterial metagenomic DNA in Vietnamese goats’ rumen and found that cellulase-producing bacteria and cellulase families were dominant. In this study, the properties of modular cellulases and the role of a FN3 in unique endoglucanase belonging to glycosyl hydorlase (GH) family 5 were determined.

**Methods:**

Based on Pfam analysis, the cellulases sequences containing FN3, Ig modules were extracted from 297 complete open reading frames (ORFs). The alkaline, thermostability, tertiary structure of deduced enzymes were predicted by AcalPred, TBI software, Phyre2 and Swiss models. Then, whole and truncated forms of a selected gene were expressed in *Escherichia coli* and purified by His-tag affinity column for assessment of FN3 ability to enhance enzyme activity, solubility and conformation.

**Results:**

From 297 complete ORFs coding for cellulases, 148 sequences containing FN3, Ig were identified. Mostly FN3 appeared in 90.9% beta-glucosidases belonging to glycosyl hydrolase family 3 (GH3) and situated downstream of catalytic domains. The Ig was found upstream of 100% endoglucanase GH9. Rarely FN3 was seen to be situated downstream of X domain and upstream of catalytic domain endoglucanase GH5. Whole enzyme (called XFN3GH5 based on modular structure) and truncate forms FN3, XFN3, FN3GH5, GH5 were cloned in pET22b (+) and pET22SUMO to be expressed in single and fusion forms with a small ubiquitin-related modifier partner (S). The FN3, SFN3 increased GH5 solubility in FN3GH5, SFN3GH5. The SFN3 partly served for GH5 conformation in SFN3GH5, increased modules interaction and enzyme-soluble substrate affinity to enhance SXFN3GH5, SFN3GH5 activities in mixtures. Both SFN3 and SXFN3 did not anchor enzyme on filter paper but exfoliate and separate cellulose chains on filter paper for enzyme hydrolysis.

**Conclusion:**

Based on these findings, the presence of FN3 module in certain cellulases was confirmed and it assisted for enzyme conformation and activity in both soluble and insoluble substrate.

## INTRODUCTION

Lignocellulases especially cellulases play crucial role in bio-economy development, thus these enzymes gained interest from many researchers all over the world for isolation of novel effective enzymes for conversion of lignocellulose into sugars [1,2].

By its structure, cellulose degrading enzymes exist in two architectures: i) have only unique catalytic domain; ii) besides the catalytic domains, many enzymes also possess additional modules which have non-catalytic activity but play an important role in enzyme function so called modularity. The main accessory modules include: a) carbohydrate-binding module (CBM); b) fibronectin3-like module (FN3) which have high diversity from 33.7% to 83.2% similarity in sequence but have homogeneous structure; c) immunoglobulin-like domain (Ig) with high similarity in conserved region; and d) functionally unknown “X” domains [3–5].

Fibronectin III presents in animal proteins with primary functions of mediating protein-protein interactions, and collocates in enzyme structure to act as a linker for protein structure stability and to get the right activity [6]. In bacteria, FN3 is found only in extracellular glycosyl hydrolase (GH) families and responsible for loosening cellulose surfaces, peeling cellulose fibers and directing cellulose chain into the catalytic core for easily converting substrates [7].

The Ig module is detected only in cellulase belonging to GH9 and plays an important role in enzyme catalysis, and thermal stability [8]. However, another recent study has shown that removing the Ig module in *Alicyclobacillus acidocaldarius* endoglucanase Cel9A results in increase both the activity and the stability of the enzyme [9]. The sequences of FN3 and Ig modules of bacterial and human enzymes are homogeneous, but their biological functions in bacterial enzymes have not been studied thoroughly [7,10].

In order to determine new cellulases for hydrolysis of cellulose biomass using modern technology, we sequenced the metagenomic DNA of bacteria in Vietnamese goats’ rumen [11]. In total, 816 genes (297 complete genes) coding for cellulases were obtained. The initial analysis has shown that the FN3 module was mainly found in the structure of beta-glucosidases (213 open reading frames [ORFs]) and endoglucanases (one ORF) structure while Ig module is only collocated in endoglucanases GH9 (30 ORFs) [11,12]. However modular structures and some characters such as pH, thermostablility of the putative enzymes containing FN3 and Ig were not illustrated. In addition, FN3 dominantly appeared only in GH3, thus we hypothesize that the FN3 module has an affinity to short polysaccharide chains and has a major role in stabilizing enzyme molecule, thus, increasing the solubility and enzyme activity. If this module is responsible for enzyme immobilization on substrate, it is extremely valuable in industry owing to the fixable, collective and reuseable ability of the enzymes.

## MATERIALS AND METHODS

### Animal care

The animal experimental protocol of this research was reviewed, discussed, and approved by the institutional ethics committee of the Institute of Genome Research Institutional Review Board (IGR IRB), Vietnam (Approval number: No. 03/QD-NCHG date 8/12/2015).

### Materials

Goat breeds Co and Bach Thao are regarded as the native goats in Vietnam. In previous studies, five goats Co and five goats Bach Thao living on natural hays in high rocky mountains at Ninh Binh and Thanh Hoa were selected and used for collecting the rumen fluids as described in previous study [11] then harvesting bacteria in the goats’ rumen. The total metagenomic DNA of the rumen bacteria was extracted and sequenced by Illumina Hiseq2500 (Illumina, San Diego, CA, USA) at BGI company in Hong Kong. The useful reads (84,625,346 reads) obtained from sequencing were assembled into 172,918 contigs by SOAPdenovo2. The contigs were revised by Rabbit tool then subjected into MetaGeneMark for prediction of open reading frames (ORFs). The function of the ORFs (164,644 ORFs) was annotated by BLAST based on Swiss-Prot, Kyoto encyclopedia of genes and genomes, non-supervised orthologous groups, cluster of orthologous groups, carbohydrate-active enzymes, and gene ontology databases. From the annotated results, 816 ORFs encoding 11 glycoside hydrolase families (GHs) of cellulases were mined [11]. In this study, the 297 complete ORFs encoding nine cellulase families were used for the prediction of cellulases modularity containing FN3, and Ig domains.

### Prediction of modularity, physical properties, the tertiary structure of cellulases

The complete ORFs encoding cellulases were subjected to the Pfam database (http://pfam.janelia.org/search) and HMM (https://www.ebi.ac.uk/Tools/hmmer) [3]. The spanning modules on enzymes were drawn by excel. Compute pI/Mw toolkit in Expasy (https://web.expasy.org/compute_pi/) was used to estimate the isoelectric point (pI) value and molecular mass of polypeptide chains. The AcalPred software [13,14] was applied for the prediction of acidic and alkaline enzymes. The thermostability of a protein was estimated by TBI software (http://www.tbi.org.tw/tools). The tertiary structure of enzymes was analyzed using two distinct tools Phyre2 (http://www.sbg.bio.ic.ac.uk/phyre2/html/page.cgi?id=index) and Swiss model (https://swissmodel.expasy.org/interactive).

### Cloning, expression, and purification of complete and truncated forms of endoglucanase XFN3GH5

**Figure 1:**
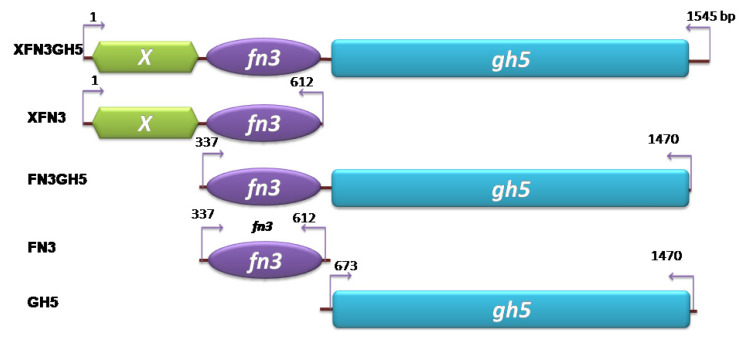
Domain structure of truncated genes *XFN3*, *FN3GH5*, *FN3*, *GH5* of *XFN3GH5* was expressed in *Escherichia coli* for investigating the function of FN3, XFN3 domains in the enzyme endoglucanase XFN3GH5. Arrows indicate the direction of polymerase chain reaction amplification. Numbers refer to orders of nucleotides. *XFN3GH5*, whole gene coding for endoglucanase containing domains X, fibronectin (FN3) and glycosyl hydrolase 5; *FN3*, *XFN3*, *FN3GH5*, *GH5* coding for truncated forms of endoglucanase XFN3GH5.

The [denovogenes] _13359 sequence (1,623 bps) encoding for endoglucanase GH5 (modular structure of the enzyme comprises domains X, fibronectin 3 and endoglucanase catalytic domain belonging to GH5, so whole mature enzyme was called as XFN3GH5) was selected to be recombinantly expressed in *Escherichia coli* (*E. coli*) for investigation of FN3 function. The sequence encoding for the mature endoglucanase (*XFN3GH5*, 1,545 bps) was codon optimized (Supplementary Table S1), artificially synthesized at Genscript (Piscataway, NJ, USA) and inserted into pET22b(+), pET22SUMO [14] at *Nco*I, *Xho*I (Figure 1). The truncated forms of the *XFN3GH5* gene including *FN3*, *GH5*, *FN3GH5* (Figure 1) was amplified by polymerase chain reaction (PCR) using primers in Table 1. The *XFN3* was amplified by primer for T7 promoter (Novagen, Madison, WI, USA) and primer fn3r (Table 1). All the truncated genes also inserted into pET22b (+), pET22 SUMO at *Nco*I, *Xho*I restriction sites for the expression of the genes in single and SUMO-fusion forms in *E. coli*. Some characteristics such as Mw, pI, of the expressed proteins were determined and indicated in Table 2.

For expression of the genes in *E. coli*, the recombinant *E. coli* strains were inoculated overnight in 5 mL Luria Bertani medium containing ampicillin (LBA) at 37°C, 200 rpm. The cultures then were diluted into optical density at 600 nm (OD_600_) of 0.1 in LBA medium and continuously incubated in the previous conditions. When OD_600_ reached 0.4 to 0.6, the cells were induced with isopropyl β-D-1-thiogalactopyranoside (IPTG) (up to 0.5 mM) and cultured for 5 hours at 25°C, 200 rpm. Some factors affecting the genes’ expression (such as media, IPTG concentration and temperature) were investigated. The cultures were then centrifuged at 6,000 rpm for 10 min and suspended in phosphate-buffered saline (PBS) 1× (one litter of PBS containing 8 g NaCl; 0.2 g KCl; 0.24 g KH_2_PO_4_; 1.42 g Na_2_HPO_4_) to OD_600_ of 10. The total expressed proteins were analyzed by sodium dodecyl sulfate-polyacrylamide gel electrophoresis (SDS-PAGE) as follows. The cell samples were mixed with 2× treatment buffer (62.5 mM Tris–HCl, pH 6.8, 2% SDS, 25% (v/v) glycerol, 0.01% bromophenol blue, 5% β-mercaptoethanol) and denatured at 95°C for 5 min then immediately kept on ice for 2 min and loaded into 12.6% SDS-polyacrylamide gel. The gel was run in BIO-RAD Mini Protean 3 Electrophoresis apparatus at the initial of 10 mA/gel during the samples immigrating in stacking gel, then increased into 20 mA/gel until the bromophenol blue has reached the lower edges of the gel. The gel was stained by staining solution (0.25% coomassie blue, 50% methanol, and 10% acetic acid) and distained by a distaining solution (50% methanol, 10% acetic acid) for 1 hour. In order to evaluate the recombinant proteins’ solubility, the cells were disrupted by sonication at 20 kHz (10s of supersonic and 20 seconds of stoppage in approximately 30 to 40 pulses) and centrifuged at 13,000 rpm for 10 minutes to separate proteins in supernatants (soluble fractions) from precipitates (insoluble fractions). The supernatants and water-suspended pellets were analyzed by SDS-PAGE.

All the recombinant proteins were purified by nickel-nitrilotriacetic acid agarose chromatography (5 mL) using phosphate buffer 50 mM without NaCl, pH 6.8 as the basic buffer. Contaminant proteins were washed with 10 column volumes of basic buffer containing 0.1 M imidazole. The target proteins were eluted by 10 column volumes of basic buffer containing 0.4 M imidazole and two-ml fractions were collected. The fractions were analyzed by 12.6% polyacrylamide gel electrophoresis.

The purified proteins were desalted using a PD-10 ultrafiltration column (GE Healthcare, Mississauga, ON, Canada) against 10% glycerol and used for functional investigation. Briefly, 500 μL of the purified proteins was applied to a PD-10 desalting column equilibrated in 10% glycerol. As soon as the samples had entered the packed bed completely, 5 mL of glycerol 10% was added gently. Fractions of 500 μL were collected and analyzed by SDS-PAGE as described above and protein concentration was measured by Bradford assay kit.

### Cellulase assay

#### Zymogram analysis

The protein samples (~50 μg in glycerol 25%) were loaded on the 7% SDS-PAGE gel containing 0.2% carboxymethyl cellulose (CMC). The electrophoresis was performed at 4°C to limit the enzyme action during immigration in the gel, at initial 10A/gel then increased to 20A/gel as described above. After electrophoresis, SDS gel was washed in distilled water for 10 minutes to remove SDS and Tris-buffer then soaked in limited volume of PBS buffer 1×, pH 6 at 37°C overnight for CMC hydrolysis by enzymes in samples. Afterwards, the gel was stained with 0.2% Congo red for 1 hour and destained with 1 M NaCl until the clear zone against red background was observed. Another duplicated SDS gel containing the same loading pattern was stained with Coomassie brilliant blue R-250.

#### Endoglucanase assay on CMC plate

The soluble fraction of recombinant proteins (0.2 mL) was dropped into prepared wells on CMC plates (1.8% agar, 0.5% CMC, pH 6). A positive control was 0.5 U cellulase (Sigma, Darmstadt, Germany) in 0.2 mL water. The plates were incubated overnight at 37°C then stained by 0.2% Congo red solution for 60 min and destained with 1 M NaCl.

#### Determination of endoglucanase activity

The enzyme activity was determined in the substrates of CMC and filter paper. Briefly, 0.75 mL of assay mixture containing 0.25 mL of 1% (w/v) of CMC or 1 mg filter paper in PBS pH 6 and 0.45 mL of diluted enzyme in PBS was incubated at 40°C for 1 hour. The enzyme reaction was stopped by adding 0.75 mL DNS reagent (1% 3,5-dinitrosalicylic acid; 1% NaOH; 18.2% K-Na-tartrate) then kept in boiling water for 15 min. Afterward, 0.25 mL K-Na-tartrate 40% was added, kept until cool at room temperature and measured at 540 nm. The enzyme activity was expressed as the amount of reducing sugar released equal to mM glucose. Each measurement was made in triplicate and the values shown in figure represented the average values of the three repeats and standard deviations. The differences between different groups of samples were canculated with Student’s t-test at a significance level of 0.05 in Microsoft Office Excel.

### The binding ability of the recombinant proteins on filter paper

The experiment investigating the ability of the SFN3 and SXFN3 binding on the filter paper surface was carried out as described by Crough et al [15] with modification. The purified SFN3, SXFN3, SFN3GH5, and SXFN3GH5 (28.6 μg each) were mixed with filter paper (1 mg) in 50 μL PBS (pH4, 6, and 8). The mixtures of SFN3+SXFN3GH5, SXFN3+ SXFN3GH5 were also prepared using the same amount of each protein. The tubes were incubated at 37°C, 150 rpm for 2 hours and centrifuged at 5,000 rpm for 5 min to obtain unbound fraction. The pellets were washed twice with PBS buffer. The remaining pellets were finally resuspended in SDS-loading buffer without the dye and boiled for 10 min to dissociate any bound proteins. The unbound, bound, and second washed proteins were analyzed by SDS-PAGE. On the other hand, the paper pellets bound with SFN3, SXFN3 after washing were used as substrates for SFN3GH5, SXFN3GH5 activities.

### The affinity of recombinant proteins with soluble carboxymethyl cellulose

This experiment was carried out as described by Kosugi et al [16] with minor modifications. Reaction mixtures (50 μL) containing 28.6 μg protein (each) including SFN3, SXFN3, SXFN3GH5, SFN3+SXFN3GH5, SXFN3+SXFN3GH5 in PBS buffer pH4 present or absent of CMC 1% were incubated at 37°C, 150 rpm for 2 hours. Glycerol was added into the aliquots of the reaction mixtures to final glycerol concentration of 25% and then samples were applied immediately to a native PAGE gel (without SDS) for electrophoresis in running buffer without SDS at the same condition described above.

### Observation by electron microscopy

For the assessment of SFN3 and the SXFN3 affect on filter paper surface, we applied the protocol described by Kataeva et al [7]. The reaction (500 μL) prepared by mixing 10 mg filter paper, 0.2 mg protein SFN3 or SXFN3, 50 μL PBS 10× (pH4) and deionized water was incubated at 37°C, 100 rpm for overnight. The samples were rinsed by distilled water then dried at 40°C. At the same time, a negative control was prepared replacing the protein with the same volume of 10% glycerol. The treated filter papers were directly secured onto scanning electron microscope (SEM). The samples were viewed at different magnifications with Jeol 6490 JED 2300 (Tokyo, Japan) and the micrographs were taken.

## RESULTS

### Distribution of FN3 and Ig modules in cellulases from goat’s rumen metagenomic data

From the metagenomic DNA data of bacteria in Vietnamese goats rumen, 816 ORFs coding for 11 cellulase GHs were mined, while 297 ORFs were complete [11]. Commonly, cellulases have a modular structure, that besides a catalytic domains the enzyme also comprises accessory domains such as CBMs, FN3, Ig. The investigation of the complete ORFs showed that 51 cellulases belonged to six GH families including GH1 (one ORF), GH8 (19 ORF), GH16 (19 ORFs), GH44 (one ORF), GH74 (1 ORF), GH94 (10 ORF) have a unique domain for catalytic function and did not contain any accessory domain. The architecture of cellulase GH3 existed in two forms: single GH3 domain (13 ORFs) and GH3 domain associated with the FN3 domain (130 ORFs). Hence, 90.9% cellulase GH3 contained FN3 module. The investigation also showed that 100% of enzymes containing GH9 domains comprised Ig (17 ORFs). While of the 82 cellulase GH5 sequences, we only found one sequence having CBM37 and one sequence containing a single FN3 domain. The reason why many cellulase GH families do not have any non-catalytic accessory domains is a mystery. With the 90.9% cellulases GH3 containing FN3 and 100% cellulase GH9 containing Ig we supposed that FN3, Ig domains must play a role for the enzymes’ activities. Interestingly, we found one endoglucanase GH5 domain (EC 3.2.1.4) was collocated with the FN3 domain.

**Figure 2:**
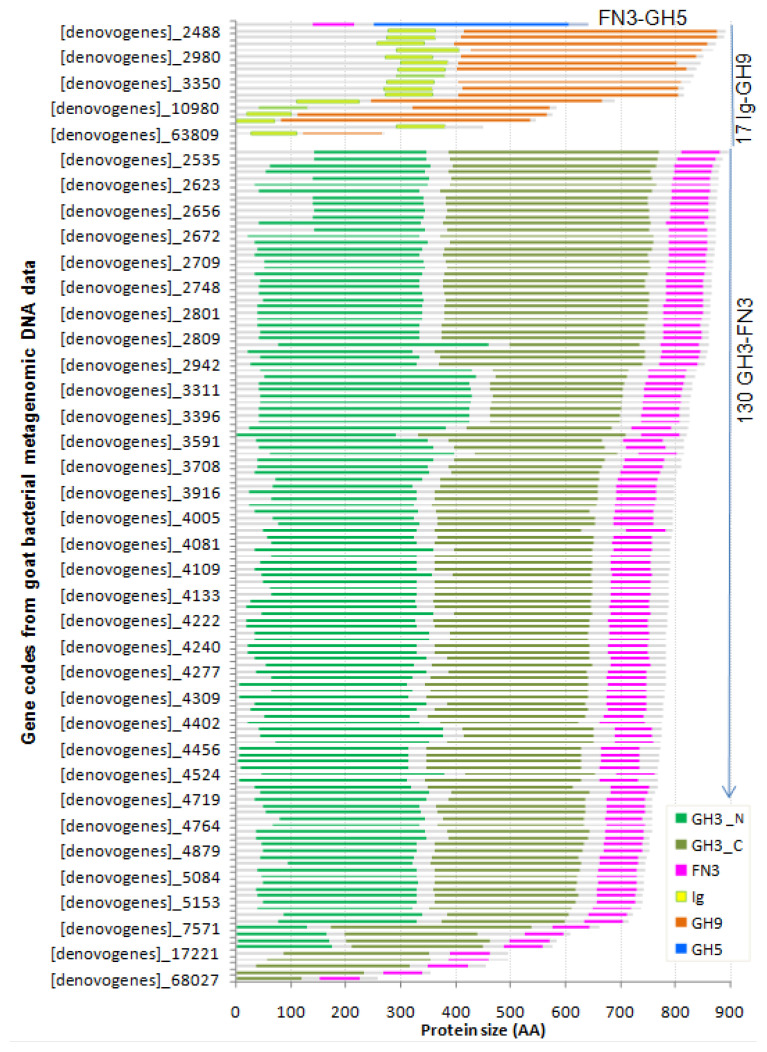
Architecture of 148 putative cellulases having FN3 or Ig domains mined from metagenomic DNA data of bacteria in Vietnam goats’ rumen. Cellulases belonging to glycosyl hydrolase (GH) family 3 (GH3) exists in two forms: single GH3 domain (13 open reading frames [ORFs]) and GH3 domain associated with the fibronectin (FN3) domain (130 ORFs). The FN3 domain was always located behind GH3_N or GH3_C domain. All enzymes containing GH9 domains (17 ORFs) comprised immunoglobulin (Ig) domain. One of the 82 cellulases GH5 contains a single FN3 domain.

Looking at the protein size of multidomain enzymes, we found that a significant part of enzymes had the size of 600 to 900 amino acids (AA). FN3 domain always located behind GH3_N or GH3_C domain, but the Ig domain always situated upstream of GH9 domain. In the modular structure of endoglucanase GH5, FN3 was not placed behind the catalytic domain as usual, instead, this domain was situated upstream of GH5 as the Ig module (Figure 2).

The results of the prediction of the alkaline enzymes showed that a 64% beta-glucosidases containing the GH3 domain were alkaline enzymes with the alkaline score higher than 0.6. There was no difference in the ratio of alkaline enzymes between groups of single module GH3 and GH3-FN3. Thus, the FN3 domain may be not related to alkaliphilic property of enzymes. In contrast, 43% endoglucanases GH5 and GH9 were alkaline enzymes (Supplementary Figure S1) especially many putative endoglucanases GH5 was extremely acid.

Thermostable enzymes are of interest for application. The investigation of the thermal stability of enzymes based on the sequences showed that half of beta glucosidases GH3 and GH3-FN3 are stable at high temperatures (the Tm higher than 65°C). There was no difference in the ratio of thermostable between single domain GH3 and GH3FN3 groups (Supplementary Figure S2). However, nearly 60% endoglucanases GH5 and FN3GH5 had Tm around 55°C to 65°C and apart served Tm lower than 55°C. The majority part of Ig-GH9 possessed Tm 55°C to 65°C. By the survey of the pI values of 143 complete sequences containing FN3 and Ig-like regions, we found only two sequences (an Ig-GH9 and an FN3-GH5) had pI higher than 9, that are cationic enzymes.

### Fibronectin 3 served the catalytic domain in soluble form in recombinant *E. coli*

**Figure 3:**
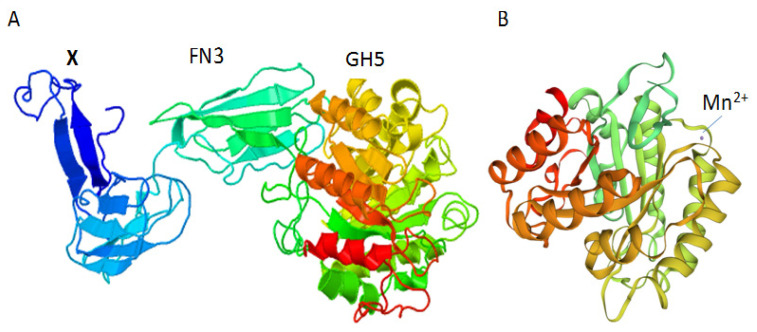
Prediction of the tertiary structure of the whole endoglucanase containing domains X, fibronectin 3 (FN3) and catalytic domain of glycosyl hydrolase 5 (GH5) by Phyre2 (A) and SwissProt (B).

The FN3 collocated with GH5 catalytic domain was uncommon, so for investigation of FN3 function, we expressed whole mature enzyme and truncated forms in *E. coli*. Based on BLASTP, the putative enzyme had the most similarity (60%) with endoglucanase code CDC67342.1. This gene was predicted from *Ruminococcus bicirculans* [12]. Using the Phyre2 tool and SwissProt, it was revealed that the protein has the highest similarity (49% in SwissProt and 47% in Phyre2) with a frame of endoglucanase 3pzt.1.A in SwissProt and c3x17B endoglucanase in Phyre2 with the confidence of 100%. SwissProt considered only in the catalytic domain and indicated that the enzyme has a ligand of Mn^++^ (Figure 3B). While Phyre2 showed the structure of the endoglucanase comprised three domains: X domain in N terminal followed by FN3 domain and catalytic domain located in the C terminal (Figure 3A). Every domain connects to the adjacent domain by a linker (Figure 3A). Secondary structure of X domain was constituted by beta sheets like FN3 module, but it shares only 51% identity in amino acid sequence with FN3 from *Ruminococcus* sp. (code SCJ53390.1).

**Figure 4:**
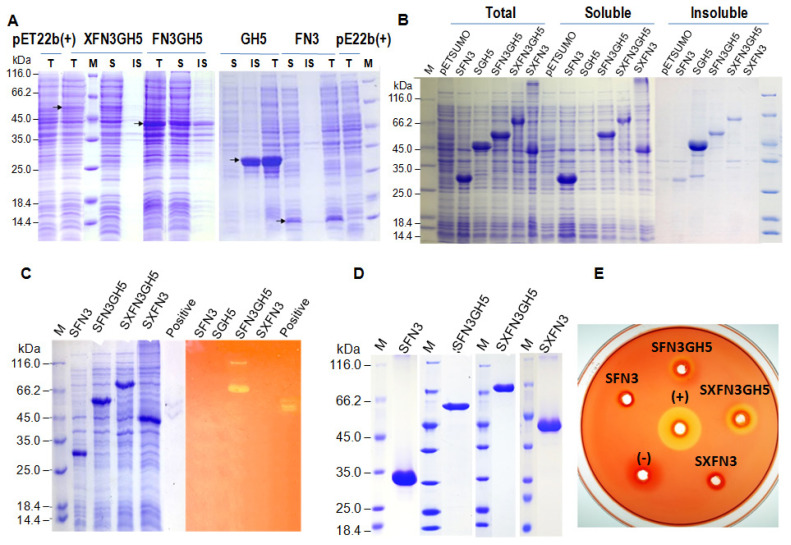
Analysis of the expression of truncated genes *XFN3*, *FN3GH5*, *FN3*, *GH5* and whole gene *XFN3GH5* coding for endoglucanase XFN3GH5 in *E. coli* in the single forms using pET22b(+) (A) and SUMO-fusion forms using pETSUMO (B), purified domains (D) by SDS-PAGE, and the activities of expressed domains by Zymogram (C) and purified proteins on CMC plate (E). (+), positive: 1U, 0.5 U cellulase (Sigma, Darmstadt, Germany) respectively. M: Standard protein (Thermo Scientific, Waltham, MA, USA). In the single form, all truncated and whole enzymes were expressed at low level, excepting domain of glycosyl hydrolase family 5 (GH5). When fusion with small ubiquitin-like modifier (SUMO) partner, the expressibility of SFN3, SXFN3, SFN3GH5, SXFN3GH5 was significant improved but the solubility of SGH5 was not improved. By Zymogram assay, only SXFN3GH5 in total proteins exhibited activity to hydrolyzing CMC. The purified SFN3GH5, SXFN3GH5 exhibited activities to hydrolyze CMC to create a clear zone by staining with Congo red in CMC-agar plate. XFN3GH5, whole mature endoglucanase containing domains X, fibronectin (FN3) and glycosyl hydrolase 5; FN3, XFN3, FN3GH5, GH5, truncated forms of of endoglucanase XFN3GH5; Prefix S, SUMO fusion; SDS-PAGE, sodium dodecyl sulfate-polyacrylamide gel electrophoresis; CMC, carboxymethyl cellulose.

For understanding the role of FN3 and XFN3 domains on enzyme activity, we cloned and expressed separated domains including FN3, XFN3, GH5, FN3GH5, XFN3GH5 in *E. coli* under the single forms using pET22b(+) and SUMO-fusion forms using pETSUMO vector [14]. The results showed that FN3, FN3GH5, XFN3GH5 were expressed at low level (Figure 4A), which may be due to high pI. The GH5 domain was expressed at a high level, but almost existed in the inclusion body (Figure 4A). In the fusion with SUMO, the expression level of proteins SFN3, SXFN3, SGH5, SFN3GH5, SXFN3GH5 was significantly improved. However, the solubility of SGH5 was not improved (Figure 4B). Although expressed in different conditions such as reducing the temperature during induction culture, using different inducer concentration, changing media, using heat shock chaperon, but GH5 and SGH5 were still in insoluble fractions. Looking at the other constructs, more than a half of FN3GH5 and SFN3GH5 were expressed in the soluble forms. Thus, FN3 must help to increase the solubility of FN3GH5 and SFN3GH5, maybe by the way to serve for protein conformation. Although SFN3GH5 presented in soluble forms, enzyme activities were not observed in Zymogram assay (Figure 4C). Whereas the X domain could not serve for increasing the solubilization of SFN3GH5, but SXFN3GH5 exhibited endoglucanase activity in the CMC substrate in Zymogram assay (Figure 4C). In the single form, the most XFN3GH5 was expressed in soluble form, but it is not evident enough for assure that the X domain helps FN3GH5 solubilization, because the expression level of XFN3GH5 was lower than FN3GH5. The decrease in translation rate can help novel protein conformation to be soluble. However, XFN3 plays a role in SXFN3GH5 conformation and stability to exhibit endoglucanase activity. The SFN3, SFN3GH5, SXFN3GH5, SXFN3 were purified successfully by His-Trap affinity column (Figure 4D). After purification, SFN3GH5 exhibited activity to hydrolyzing CMC to create a clear zone by staining with Congo red (Figure 4E).

### The SFN3 and SXFN3 increased activities of SFN3GH5, SXFN3GH5 to hydrolysis of carboxymethyl cellulose and filter paper

**Figure 5:**
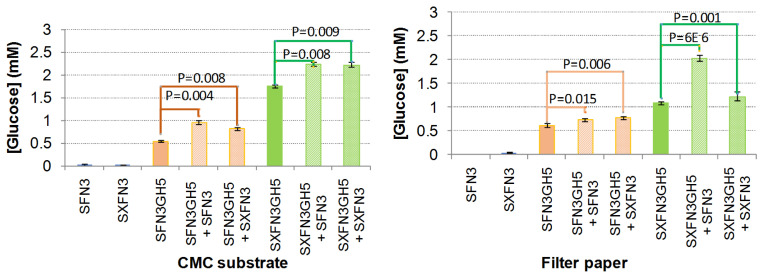
Hydrolysis activities of truncated forms of the SXFN3GH5 and combinations of catalytic domains with SFN3, SXFN3 domains in CMC and filter paper. The values shown in the figure represented the average values of the three repeats and standard deviations. The differences between different groups of samples were canculated with Student’s t-test at a significance level of 0.05 in Microsoft Office Excel. The SFN3, SXFN3 incereased endoglucanase SFN3GH5, SXFN3GH5 activities in CMC and filter paper. SXFN3EG5, whole mature endoglucanase containing domains X, fibronectin (FN3) and glycosyl hydrolase 5 expressed under fusion form with small ubiqutin-like modifier (SUMO); SFN3, SXFN3, SFN3GH5, SGH5; truncated forms of SXFN3GH5; CMC, carboxymethyl cellulose.

The truncated forms of SFN3, SXFN3, SFN3GH5, SXFN3GH5 differed in their hydrolysis activities in CMC and filter paper. In which, SFN3 and SXFN3 did not exhibit catalytic activity, whereas SXFN3GH5 produced three and two times more reducing sugars from CMC and filter paper, respectively, than SFN3GH5 alone. Mixing of SFN3, SXFN3 with SFN3GH5, SXFN3GH5 respectively, resulted in a significant increase in reducing sugars production from CMC. However, in filter paper, both SFN3, SXFN3 did not affect the hydrolysis rate of SFN3GH5, while SXFN3 increased activities of SXFN3GH5 slightly. The SFN3 promoted the hydrolysis rate of SXFN3GH5 significantly on this substrate (Figure 5). The result showed that SFN3 and SXFN3 have a more positive effect on the enzyme activities not only when these domains were directly attached to the catalytic domain, but also when they were simply mixed with them.

### The SFN3 and SXFN3 promotes filter paper hydrolysis by modifying substrate surface

Filter papers comprise of crystalline cellulose beside free cellulose fibers. Thus, the domain FN3 and XFN3 increased SFN3GH5 and SXFN3GH5 activities by two hypothesized reasons: i) these modules modified filter paper surface for enzyme accessing into cellulose fibers then hydrolysis; ii) these modules increased affinity of SFN3GH5 and SXFN3GH5 on the filter paper.

**Figure 6:**
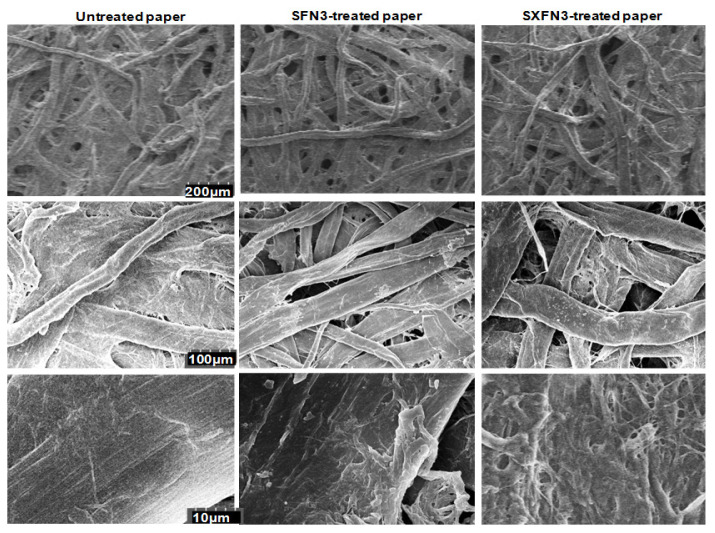
Scanning electron micrographs of filter papers untreated and treated with purified recombinant SFN3, SXFN3 (columns) of endoglucanase SXFN3GH5 at different magnifications (rows). The SFN3 and SXFN3 modified the surface morphology of filter paper and loosened cellulose fibers on the paper surface. SFN3, small ubiquitin-like modifier fused with fibronectin 3; SXFN3, SFN3 fused with X domain; SXFN3GH5, whole endoglucanase.

To answer the first hypothesis, the filter paper was treated with SFN3, SXFN3 then scanned by electron microscopy (Figure 6). As a result, there were changes in the surface morphology of native filter paper and filter paper treated with the recombinant domains. The surface of the control paper at the lowest magnification was relatively smooth, and slightly exfoliated when observed at the higher magnifications. On the contrary, the surface of the SFN3 and SXFN3-treated papers was rough even at the lowest magnification and crenellated when surveyed at the higher magnifications. Many loosened cellulose fibers and underlying microfibrils were seen in the SFN3, SXFN3-treated papers.

With the aim to assess the ability of SFN3 and SXFN3 in increasing affinity of SFN3GH5 and SXFN3GH5 to the filter paper, we checked the fractions of bound, unbound and washed proteins including SFN3, SXFN3, SXFN3GH5, SFN3+ SXFN3GH5, SXFN3+SXFN3GH5 from their mixtures with filter papers. Unfortunately, we found a few proteins in bound fractions. Temperature (20°C, 40°C, and 60°C) and pH (4, 6, and 8) did not affect the binding of the proteins with the filter paper. The SFN3 and SXFN3 did not enhance SXFN3GH5 binding on the filter papers in the mixtures (results are not shown). These results mean that SXFN3 or SFN3 did not help enzyme to anchor to the substrate. After washing all the unbound proteins, the hydrolysis activity of SFN3GH5 did not increase in the filter papers treated with SXFN3 or SFN3 but the activity of SXFN3GH5 slightly increased. This indicated that SXFN3 or SFN3 might help to increase the affinity of SXFN3GH5 to filter paper.

### Binding affinity of the recombinant proteins for carboxymethyl cellulose

**Figure 7:**
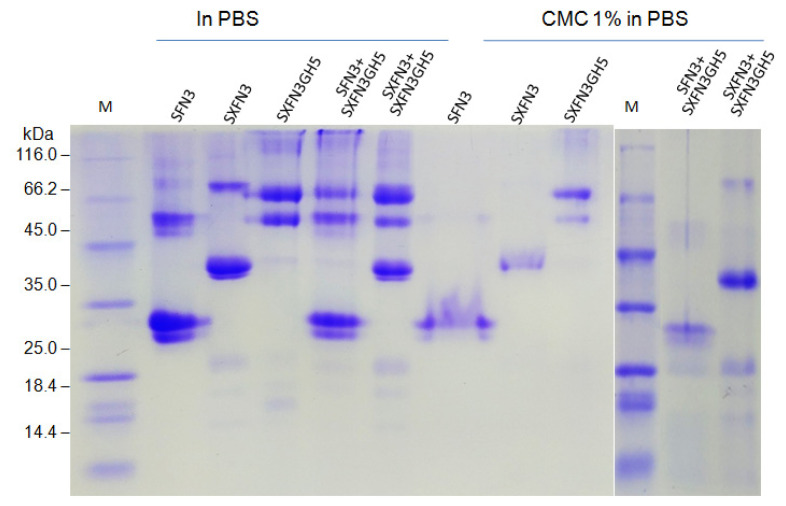
Analysis of polymerization ability of truncated (SFN3, SXFN3) and whole forms (SXFN3GH5) in the present or absent of CMC 1% by electrophoresis in 12.6% native polyacrylamide gel. The purified SFN3, SXFN3, SXFN3GH5 existed in both monomer and polymer forms with different molecular weights. The SFN3, SXFN3, SXFN3GH5 in the mixtures SFN3+SXFN3GH5, SXFN3+SXFN3GH5 were more polymerized than in the single proteins. In the presence of CMC, all the single proteins or mixture of the proteins were effectively bound to CMC to increase molecular weight. SFN3, small ubiquitin-like modifier fused with fibronectin 3; SXFN3, SFN3 fused with X domain; SXFN3GH5, SXFN3 fused with glycosyl hydolase 5; CMC, carboxymethyl cellulose; PBS, phosphate-buffered saline.

To confirm the positive effect of SFN3, SXFN3 on SXFN3GH5 activity related to increase in enzyme affinity to the substrate, we examined whether SFN3, SXFN3 could bind or interact with the mature enzyme SXFN3GH5; and SFN3, SXFN3, SXFN3GH5 could bind to soluble CMC; and whether SFN3, SXFN3 could increase the binding ability of SXFN3GH5 with CMC. As a result, in native PAGE gel, the purified SFN3, SXFN3, SXFN3GH5 existed in both monomer and polymer forms with different molecular weights. Comparing bands of SFN3, SXFN3, SXFN3GH5 and the corresponding bands in the mixtures SFN3+SXFN3GH5, SXFN3+SXFN3GH5 we found that SFN3, SXFN3, SXFN3GH5 in the mixtures were more polymerized than in the single proteins. In the mixtures, SFN3, SXFN3GH5 were polymerized much more than SXFN3, SXFN3GH5. This result revealed that both SFN3, SXFN3 interacted with SXFN3GH5 to increase their polymerization or bound to SXFN3GH5 to make different quaternary structures. However, the positive effect of SFN3 was stronger than of SXFN3 (Figure 7). It is the reason why SFN3 increased the catalytic activity of SXFN3GH5 better than SXFN3.

In the presence of CMC, all the single proteins or mixture of proteins were effectively bound to CMC to increase protein molecular weight. We also observed that this binding was much stronger in the presence of both SFN3 and SXFN3GH5, and a little bit stronger in the present of both SXFN3 and SXFN3GH5. This confirmed that SFN3 increased affinity between enzyme and substrate better than SXFN3 (Figure 7).

## DISCUSSION

Cellulases comprise endoglucanases (EC 3.2.1.4) randomly hydrolyzing the cellulose chains into shorter polysaccharides, cellobiohydrolases (EC 3.2.1.91) hydrolyzing beta-1,4 glucosidic bond from the non-reducing end (CHB I) or reducing end (CBH II) to release cellobioses and betaglucosidases (EC 3.2.1.21) digesting cellobioses into glucoses. According to CAZy database, cellulases contain 22 GH families. However, endoglucanase GH9 is considered the most conserved cellulase and widely distributed among Prokaryotes and Eukaryotes. The GH5 is known to be the largest family. In this study, GH3 coding for beta glucosidase was the most abundant (134 ORFs), followed by GH5 (82 ORFs) and then GH9 (17 ORFs). Among the modular cellulases, 100% endoglucanases GH9 have Ig domain. Many previous studies shown that bacterial endoglucanase GH9 has a modular structure, usually contains Ig and Ig is located upstream of GH9 [8,17,18]. Ig domains were shown to be essential for the activity of the enzyme [19]. The deletion of an Ig domain led to complete inactivation of CbhA cellobiohydrolase from *Clostridium thermocellum* [10].

It is rather rare to find endoglucanase GH5 collocated with the FN3 module. For examples, Taylo et al [20] screened more than 20,000 modules of diverse endoglucanase GH5 from marine bacterium *Saccharophagus degradans* and found there is no other enzyme located with FN3 module. The FN3 was found in CelA GH9, GH48 [21] and responsible for loosening cellulose surfaces, peeling cellulose fibers for the enzyme access and hydrolysis [7]. The family GH5 comprises of 51 subfamilies [22]. GH5 members are commonly encoded as parts of multi-modular polypeptide chains containing other catalytic, substrate-binding and functionally unidentified modules. However, endoglucanase GH5 (EC 3.2.1.4) has nine subfamilies: GH5-1, GH5-2, GH5-4. GH5-5, GH5-22, GH5-25, GH2-26, GH5-37, GH5-39 and are the key endoglucanases in lignocellulose degradation and used in industrial enzyme cocktails to break down lignocellulose biomass. However, the FN3 module is usually present with GH5-8 (EC 3.2.1.78), GH5-34 (EC 3.2.1.-) and CBM also, and is located randomly at the N- or C terminal of the catalytic domain, but it is rare to see in endoglucanase GH5 [22]. From our knowledge, this is a rare case, with FN3 placed in the front of the catalytic domain of endoglucanase GH5 (EC 3.2.1.4).

In agreement with this study, besides family GH5, the FN3 module was found only in bacterial cellulase that was situated adjacent in C-terminal or downstream of other catalytic domains including GH3, GH6, GH9, GH48 [7,23–27], especially in GH3. Fibronectins are generally considered to function as bacterial adhesins. With 90.9% ORFs coding for GH3 containing FN3, FN3 must play an important role for this enzyme function. But endoglucanase GH5 containing FN3 is an uncommon case, we supposed that this domain might assist enzyme binding to the substrate and promote cellulose hydrolysis. Thus, we expressed the whole gene and truncated forms to elucidate the function of the FN3 module in this enzyme.

The endoglucanases active at high pH are good candidates for biofuel and detergent industry. Beta-glucosidase has great application prospects in many industrial fields, including medicine, textiles, chemical engineering, paper engineering, food and fermentation industries, wastewater treatment, and in feed additives. Some chemical processes in these fields are carried out under strongly alkaline conditions. Therefore, the identification of alkaline-active β-glucosidase is particularly promising for application in the above settings. In this study, we used AcalPred software for the prediction of alkaline or acidic enzymes. Accordingly, the probability score ranges from 0 to 1, which corresponds to the active pH of enzymes from extremely acid to alkali. Here 64% beta-glucosidases GH3 are alkaline enzymes with an alkaline score higher than 0.6 and many beta-glucosidases codes have an alkaline score higher than 0.95. The SXFN3GH3 was predicted as an acidic enzyme with an alkaline score of 0.08. The experimental result showed that the optimal pH for the enzyme hydrolyzing CMC was 4. Thermostable β-glucosidases have been reported to exhibit great potential for use in industries such as in food processing and bioconversion of lignocellulolytic biomasses into fermentable sugars for energy generation as they decrease the amount of enzyme needed and remain undenatured under elongated hydrolysis conditions. Here, nearly 50% beta-glucosidases GH3, 21% endoglucanases GH5, 18% endoglucanases GH9 were predicted to be thermostable at ≥65°C. In the case of SXFN3GH3, this enzyme was predicted to be stable at the temperature lower than 55°C and the experimental result showed that the optimal temperature for the enzyme hydrolyzing CMC was 40°C. Storage of enzyme at 60°C, 30 min led to a 50% loss in enzyme activity. Thus, this is a valuable source for mining alkaline and thermostable enzymes.

In this study, we also investigated the impact of FN3 module on the enzyme activity. We found that the deletion of the FN3 module led to the expression of the catalytic domain in the insoluble form without activity on the CMC substrate. Hence, the abolishment of the enzyme activity related to the lack of proper structure of the catalytic domain although FN3, XFN3 and catalytic domain GH5 are independently structured at secondary and tertiary architecture. The FN3 only helped for better conformation of the catalytic domain, but for the proper and stable structure of the catalytic domain, both X and FN3 modules are required. Thus, XFN3 may interact with the catalytic domain to keep the structure stable. Zhou et al [28] have shown that the deletion of the FN3 module in *Thermobifida fusca* Cel9A reduced 43% enzyme activity on cellulose when compared to the wild-type, but the authors have not explained a reason for the activity reduction.

In this study, FN3 played a role in the solubilization of catalytic domain when it was attached to the catalytic domain and partly helps facilitating enzyme activity. Hence this domain may assist the conformation of the catalytic domain to proper structure for both enzyme solubility and activity. However, there is no research indicating the role of FN3 for solubilization of catalytic domain, but a previous study revealed the role of Ig domain in conformation of the active site and prevention of catalytic domain aggregation [8]. At the same amount of protein, SXFN3GH5 exhibited three times higher activity than SFN3GH3 in the CMC substrate. Thus, XFN3 played an important role in enzyme conformation. On the other hand, in the mixture, both SFN3 and XFN3 increased the endoglucanase activities of SFN3GH5 and SXFN3GH5. The positive effect of FN3 from *Clotridium thermocellum* cellobiohydrolase CbhA is also observed to increase the activity of recombinant endoglucanase CelE originated from *Orpinomyces* in the mixture, while this FN3 did not significantly increase the activity of the CbhA in a mixture [7]. Other study indicated that modules display more prominent interactions inside their structural units than with other parts of the modular enzymes, they use relatively independent folds thus they preserve individually their folds and biological functions when expressed in heterologous host strains [29].

Our investigation showed that the FN3 domain could modify the filter paper by loosening cellulose surface, peeling cellulose fiber observed by SEM, thus enable enzyme accessing cellulose for hydrolysis. This function of the FN3 module is also seen in the case of *C. thermocellum* cellobiohydrolase CbhA to exfoliate and separate cellulose chains to expose additional sites of cellulose for hydrolysis by catalytic domain [7].

A previous study has indicated that the presence of the FN3 domain beside catalytic domain does not help in recognition of small substrates but involves the anchoring of the enzyme on large polymeric substrates such as lignin [30]. The adsorption of the FN3 on lignin requires positive net charge and temperature independence. In this study, the adsorption/binding of the FN3 on filter paper also did not depend on the pH and temperature. However, FN3 was not seen to anchor SXFN3GH5 on filter paper because the main fraction of SFN3, SXFN3, SXFN3GH5, SFN3GH5 were rinsed from filter paper after 2 hours implementing reaction at 37°C. Recently, Lima et al [30] suggested that FN3 domain from *Aspergillus niger* beta-glucosidase absorbs on lignin through numerous arginine residues scattered on the FN3 surface. However, in this study, we found that the FN3 increased the affinity of enzyme to the soluble substrate as CMC and enhanced the proteins interactions.

## Figures and Tables

**Table 1:** Primers used to amplify *FN3*, *GH5* genes of the gene *XFN3GH5* encoding mature endoglucanase XFN3GH5

Primers	Sequence (5′-3′)
Fn3f	TCCATGGCGACCAACCCGGCGAAGGTTAC
Fn3r	TCTCGAGGGTCTTAACGGTCACGTTATCGC
Gh5f	TCCATGGTTAACCAGAGCGGTCAAATTTTC
Gh5r	TCTCGAGGTCGGTGCCGGTCTTAAACG

Underlined sequences indicated restriction sites for cloning gene into pET22b(+) and pETSUMO.

**Table 2:** Estimated characters of the truncated proteins (FN3, XFN3, GH5, FN5GH5) of endoglucanase XFN3GH5 expressed in *Escherichia coli*

Name of gene fragment	Description	Non fusion with SUMO				In fusion with SUMO			
	
		Protein name	Size (bp)	pI	MW (kDa)	Protein name	AA	pI	Mw (kDa)
*FN3*	Fibronectin 3	FN3	276	9.79	9.8	SFN3	201	8.90	22.9
*XFN3*	XFN3	XFN3	612	10.1	22.0	SXFN3	316	9.62	35.1
*GH5*	Endoglucanase	GH5	798	8.52	29.7	SEGH5	378	7.29	42.7
*FN3GH5*	Fibronectin 3-endoglucanase	FN3GH5	1,134	9.26	41.5	SFN3GH5	487	8.95	54.5
*XFN3GH5*	Whole gene	XFN3GH5	1,545	9.64	59.0	SXFN3GH5	627	9.37	69.5

SUMO, mall ubiquitin-like modifier; pI, isoelectric point; MW, molecular weight.
