# Supplementary material for: Some characters of bacterial cellulases in goats’ rumen elucidated by metagenomic DNA analysis and the role of fibronectin 3 module for endoglucanase function
Source: Anim Biosci. 2020 Aug 21;34(5):867–79. doi: 10.5713/ajas.20.0115 (PMC8100471; doi:10.5713/ajas.20.0115)
Supplement: Supplementary file 1 [file ajas-20-0115-suppl.pdf]

Supporting information

TABLE S1. The sequence of the *xfn3gh5* gene. Italic characters coding for X domain; shaded sequence is for FN3 module; underlined sequence is catalytic domain GH5

ccatg ***gcg gtg ccg gtt ggt agc ggt gac aac gcg gtg att aac agc att acc gcg gag gcg***
M A V P V G S G D N A V I N S I T A E A
gcg acc gtg agc gtt ccg aag gtg acc ggt ctg aaa agc cag acc ctg ggc acc gat aag
A T V S V P K V T G L K S Q T L G T D K
gtt aaa ctg acc tgg aac aag att agc aac gcg acc ggt tac cag gtg ttc atg cag aag
V K L T W N K I S N A T G Y Q V F M Q K
aac ggc gtt tat acc cgt ctg att agc atc tac agc aac acc aac agc tat acc gtg agc
N G V Y T R L I S I Y S N T N S Y T V S
aac ctg ccg gcg gcg cgt atc acc aac ctg aaa gtg cgt gcg tgg cgt acc gtt ggt ggc
N L P A A R I T N L K V R A W R T V G G
aaa aac tac ttt ggt ccg ctg agc gcg gcg cgt acc acc gcg acc aac ccg gcg aag gtt
K N Y F G P L S A A R T T A T N P A K V
acc aac att cag agc acc gcg ggc agc acc agc atc aag ttt acc tgg aac aaa gtg aac
T N I Q S T A G S T S I K F T W N K V N
tgc agc tac tat cgt att tac gtt aag caa gcg gac ggc aag tgg aaa ggc ctg aaa aac
C S Y Y R I Y V K Q A D G K W K G L K N
agc acc acc aac agc gtg acc gtt acc ggt ctg aac gcg aac acc agc tat acc ttt tgc
S T T N S V T V T G L N A N T S Y T F C
gtg cgt gcg tgg caa acc gac tgg agc aag aaa gat cac ggc agc gtt ctg agc gat aac
V R A W Q T D W S K K D H G S V L S D N
gtg acc gtt aag acc aaa acc gcg gcg acc ccg gtg gcg gcg aac ggt cgt ctg agc gtg
V T V K T K T A A T P V A A N G R L S V
aag ggc gcg aac atc gtt aac cag agc ggt caa att ttc aag atc aag ggt atg agc acc
K G A N I V N Q S G Q I F K I K G M S T
cac ggc att atg tgg gag gac ttt cgt aac atc ctg agc tac aac agc ctg aag gtt ctg
H G I M W E D F R N I L S Y N S L K V L
cgt gac gat tgg aaa tgc aac acc att cgt atc gcg atg tat acc gcg gag tgg ggt ggc
R D D W K C N T I R I A M Y T A E W G G
tac acc acc ggt agc acc tat gcg gcg cag gcg aag gcg cgt gtg aaa gcg ggt gtt gaa
Y T T G S T Y A A Q A K A R V K A G V E
aac gcg aaa agc gcg ggc atg tac gcg atc att gac tgg cac atc ctg agc gac ggt gat
N A K S A G M Y A I I D W H I L S D G D
ccg cgt acc cac cag agc caa gcg gtt gcg ttc ttt aag gaa atg gcg aac acc tac aag
P R T H Q S Q A V A F F K E M A N T Y K
aac tac gat aac gtg atc tac gaa atc tgc aac gaa ccg aac ggt ggc gtt acc tgg aac
N Y D N V I Y E I C N E P N G G V T W N
ggt ggc att aag agc tac tgc cag gcg gtg gtt aac gcg atc cgt caa tat gat aaa aac
G G I K S Y C Q A V V N A I R Q Y D K N
gcg atc att atc tgc ggc acc ggc acc tgg agc caa gac atc gat aag gtg ctg ggt aac
A I I I C G T G T W S Q D I D K V L G N
cgt ctg agc gac aaa aac tgc gtt tac gcg ctg cac ttc tat gcg aac acc cac acc gat
R L S D K N C V Y A L H F Y A N T H T D
tgg ctg cgt agc cgt ctg aag agc tgc tac aac agc ggc ctg ccg gtg ctg gtt agc gag
W L R S R L K S C Y N S G L P V L V S E
ttc ggc acc tgc gat gcg agc ggt aac ggt ggc ttt aac aag tac cag acc caa gaa tgg
F G T C D A S G N G G F N K Y Q T Q E W
ctg aag ctg tgc gat agc ctg aaa gtg ggt tat att aac tgg agc gcg gcg aac aaa agc
L K L C D S L K V G Y I N W S A A N K S
gaa acc gcg agc gcg ttt aag acc ggc acc gac ctg aaa agc att acc gcg ggc acc agc
E T A S A F K T G T D L K S I T A G T S
cag ctg acc gaa agc ggc aaa ctg gtt cgt gat tgg tat cgt gcg cac ctc gag
Q L T E S G K L V R D W Y R A H L E

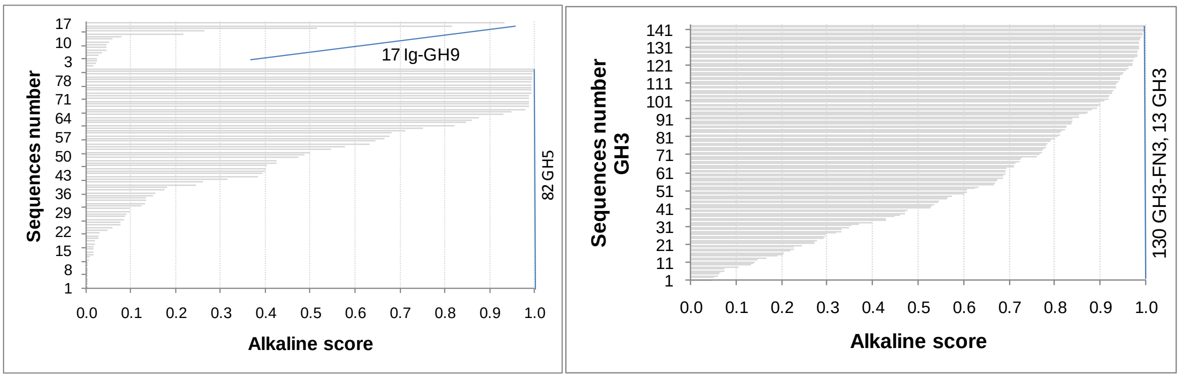

Figure S1. Prediction of alkaline enzymes GH3, GH5, GH9 based on sequences

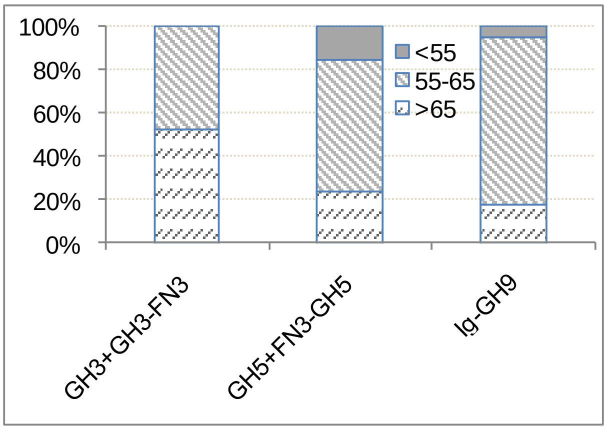

Figure S2. Prediction of the temperature melting of putative cellulase GH3, GH3-FN3, GH5, FN3-GH5, Ig-GH9 based on amino acid sequence
